# Purinergic Signaling in Gut Inflammation: The Role of Connexins and Pannexins

**DOI:** 10.3389/fnins.2016.00311

**Published:** 2016-06-29

**Authors:** Erica F. Diezmos, Paul P. Bertrand, Lu Liu

**Affiliations:** ^1^School of Medical Sciences, University of New South WalesSydney, NSW, Australia; ^2^School of Medical Sciences, RMIT UniversityBundoora, VIC, Australia

**Keywords:** purinergic receptors, connexins, pannexins, inflammatory bowel disease, gastrointestinal inflammation

## Abstract

Purinergic receptors play an important role in inflammation, and can be activated by ATP released via pannexin channels and/or connexin hemichannels. The purinergic P2X7 receptor (P2X7R) is of interest since it is involved in apoptosis when activated. Most studies focus on the influence of pannexin-1 (Panx1) and connexin 43 (Cx43) on ATP release and how it affects P2X7R function during inflammation. Inflammatory bowel disease (IBD) is characterized by uncontrolled inflammation within the gastrointestinal system. At present, the pathophysiology of this disease remains largely unknown but it may involve the interplay between P2X7R, Panx1, and Cx43. There are two main types of IBD, ulcerative colitis and Crohn's disease, that are classified by their location and frequency of inflammation. Current research suggests that alterations to normal functioning of innate and adaptive immunity may be a factor in disease progression. The involvement of purinergic receptors, connexins, and pannexins in IBD is a relatively novel notion in the context of gastrointestinal inflammation, and has been explored by various research groups. Thus, the present review focuses on the current research involving connexins, pannexins, and purinergic receptors within the gut and enteric nervous system, and will examine their involvement in inflammation and the pathophysiology of IBD.

## Introduction

Extracellular ATP can act on purinergic receptors in the gastrointestinal (GI) system to mediate a variety of actions depending on the receptor type and localization (Surprenant and North, 2009; Burnstock, 2014; Ochoa-Cortes et al., 2014). ATP is involved in excitatory neurotransmission within the enteric nervous system (ENS) via P2X receptors (P2XR) and P2Y receptors (P2YR) (Burnstock and Williams, 2000; Monro et al., 2004; Gallego et al., 2006, 2008; Ren and Bertrand, 2008). ATP acts as both an autocrine and paracrine molecule, altering ion transport, cell-cell communication, and inflammation (Burnstock and Williams, 2000; Boisse et al., 2009; Corriden and Insel, 2010; Junger, 2011; Roberts et al., 2012). Among the various types of purinergic receptors, the P2X7R is of particular interest as its activation promotes inflammation by increasing inflammatory cytokine release from immune cells in the presence of stimuli such as lipopolysaccharide (Bianco et al., 2005; Pelegrin and Surprenant, 2006; Surprenant and North, 2009; Idzko et al., 2014). The involvement of purinergic receptors in the pathophysiology of inflammatory diseases is a recurring theme and has been studied in the context of inflammatory bowel disease (IBD) in conjunction with exploring the mechanisms of ATP release. More recently, studies have focused on the involvement of two families of protein channels that have been shown to mediate ATP release extracellularly: the gap junction family of connexin channels, and the more novel pannexin channels.

Connexins are known for forming gap junctions between two adjacent cells, but can also form unopposed hemichannels that allow small hydrophilic molecules such as nucleotides and ions, to pass across the cellular bilayer (Vinken et al., 2010). Connexin (Cx) subtypes are classified according to their molecular weight and certain types of connexin hemichannels such as Cx43 may be involved in extracellular release of ATP (Fortes et al., 2004; Kang et al., 2008; Wang et al., 2013a; Csoka et al., 2015; Brown et al., 2016). Pannexin channels are structurally similar to connexin hemichannels, with both being made up of six subunits that exist either in homomeric (consisted of the same subunits) or heteromeric (made up of different subunits) states (D'Hondt et al., 2009). However, connexins and pannexins do not share sequence homology and thus are genetically unrelated (Baranova et al., 2004). There are three types of pannexins that differ at the N and C termini of their subunits: pannexin-1 (Panx1), pannexin-2 (Panx2), and pannexin-3 (Panx3) (Baranova et al., 2004). Panx1 is ubiquitous and the most well-studied in the literature. Similar to connexins, many studies have provided evidence to support a role for pannexins as ATP release channels in various systems (Schenk et al., 2008; Ransford et al., 2009; Woehrle et al., 2010a; Junger, 2011; Xia et al., 2012; Orellana et al., 2013; Beckel et al., 2014). Both pannexin channels and connexin hemichannels are thought to act as “ATP release channels” or conduits for ATP transport from the cell cytosol to the extracellular fluid (Locovei et al., 2006a; Lohman and Isakson, 2014). Panx1 and Cx43 channels have been shown to open under a variety of conditions, for example, after activation of purinergic receptors, mechanical stress or altered levels of intracellular Ca^2+^ (Bao et al., 2004; Locovei et al., 2006b; Burra and Jiang, 2009; De Vuyst et al., 2009). Channel opening is most likely regulated by elevated levels of extracellular ATP (Qiu and Dahl, 2009; Lohman and Isakson, 2014).

The present review will focus on current research involving purinergic receptors, connexins, and pannexins within the gut and the ENS, with a focus on their role during inflammation. The review will also explore their roles in models of inflammation in other organ systems to provide an insight on possible mechanisms of etiology in IBD.

## Purinergic receptors and their involvement in inflammation

### Subtypes of purinergic receptors

Purinergic P2 receptors are classified into two main categories: the P2XR and the P2YR. P2XR are ATP-gated trimeric ion channels, and P2YR exist as G-protein coupled receptors (Ralevic and Burnstock, 1998; von Kugelgen and Harden, 2011). Both receptors are further classified into their subtypes. P2XR are numbered from 1 to 7 (P2X1-P2X7) (Ralevic and Burnstock, 1998). P2YR subtypes are divided into P2Y1-like receptors (P2Y1, P2Y2, P2Y4, P2Y6, P2Y11) and P2Y12-like receptors (P2Y12, P2Y13, P2Y14) where only P2Y2 and P2Y11 have ATP as their main endogenous ligand (von Kugelgen and Harden, 2011). P2X7R are of considerable interest in inflammation since this is the only subtype that is resistant to desensitization (North, 2002). Thus, it can be activated for a prolonged period of time and can function in positive feedback signaling that is observed in events such as apoptosis (North, 2002; Locovei et al., 2007; Kurashima et al., 2012; Idzko et al., 2014; Kuhny et al., 2014).

### Expression of P2X receptors in the intestine

All of the P2XR have been detected in the mammalian gut, however, not all receptor subtypes have established roles. P2X1R are found at sympathetically innervated smooth muscles and function as neurotransmitter receptors where they exist as homomeric ATP gated ion channels (Ralevic and Burnstock, 1998; Surprenant and North, 2009). P2X2R are present in the myenteric ganglia of the ENS and are most likely expressed in intrinsic primary afferent neurons, inhibitory motor neurons, non-cholinergic secretomotor neurons, and on vagal afferent nerve endings in the stomach (Castelucci et al., 2002). P2X3R are present on cholinergic secretomotor neurons, ascending interneurons, and on both excitatory and inhibitory motor neurons (Poole et al., 2002). P2X4R are expressed in the rat pyloric sphincter, rat intestinal crypts, parotid glands, and salivary glands (Tanaka et al., 1996; Tenneti et al., 1998). P2X5R are found on canine longitudinal muscle, enteric ganglia on mouse intestine, and on interstitial cells of Cajal in guinea pig intestine (Ruan and Burnstock, 2005). P2X6R mRNA was only found in rat bile duct (Doctor et al., 2005). In contrast, the P2X7R is expressed throughout the intestine in a variety of tissue types including intestinal epithelial cells, mast cells, macrophages, and lymphocytes (Martin et al., 1998; Ralevic and Burnstock, 1998; Pelegrin et al., 2008; Cesaro et al., 2010; Idzko et al., 2014; Kuhny et al., 2014; Shoji et al., 2014). The P2X7R is also expressed in the enteric ganglia of rodents where it was found localized to intrinsic primary afferent neurons, inhibitory motor neurons and glial cells (Vanderwinden et al., 2003; Cesaro et al., 2010; de Campos et al., 2012; Gulbransen et al., 2012; da Silva et al., 2015). Table 1 summarizes P2XR localization in the intestine.

**Table 1:** **Localization of P2X receptors in the intestine**.

**Receptor**	**Localization in the gastrointestinal system**
P2X1R	Sympathetically innervated smooth muscles
P2X2R	Intrinsic primary afferent neurons, inhibitory motor neurons, non-cholinergic secretor motor neurons, and on vagal afferent nerve endings in the stomach
P2X3R	Cholinergic secretomotor neurons, ascending interneurons, excitatory and inhibitory motor neurons
P2X4R	Rat pyloric sphincter, rat intestinal crypts, parotid glands, and salivary glands
P2X5R	Canine longitudinal muscle, mouse enteric ganglia, and guinea pig interstitial cells of Cajal
P2X6R	Bile duct
P2X7R	Epithelial cells, mast cells, macrophages, and lymphocytes
	Enteric ganglia expression: intrinsic primary afferent neurons, inhibitory motor neurons, and glial cells

### P2X7 receptors in inflammatory bowel disease

Recent studies have suggested roles for P2X7R in areas such as cell proliferation, tumor growth, and neural cell function (Vanderwinden et al., 2003; Adinolfi et al., 2012; Rigato et al., 2012; Bartlett et al., 2014; Hofman et al., 2015). However, the P2X7R, when activated by ATP, is best established as an important player in inflammation and apoptosis as it has been shown to enable pro-inflammatory cytokine release from immune cells (Bianco et al., 2005; Pelegrin and Surprenant, 2006; Idzko et al., 2014). In addition to its role in the inflammatory response, the P2X7R has been explored in the context of IBD, which has the characteristics of inflammation and dysmotility (Locovei et al., 2006b, 2007; Pelegrin and Surprenant, 2009; Gulbransen et al., 2012; Roberts et al., 2012; Antonioli et al., 2014). In one clinical study, patients with Crohn's Disease (CD) were observed to have a higher expression of P2X7R and higher apoptotic rates in the mucosa layer of the colon (Neves et al., 2014). Most studies of P2X7R in animal models of colitis that have attempted to counteract the alterations in P2X7R expression have concluded that there is a significant role for P2X7R. In one study of a rat colitis model induced by trinitrobenzene sulfonic acid (TNBS), blockade of the P2X7R resulted in the reduction of T-cell and macrophage infiltration in the lamina propria and a decrease in the overall severity of inflammation (Marques et al., 2014). In another preclinical study, P2X7R^−∕−^ mice subjected to TBNS or dextran sulfate sodium (DSS) failed to develop inflammation or other symptoms associated with colitis, implying an important role for P2X7R in the inflammatory process (Neves et al., 2014). A recent study using a TNBS rat model of experimental ulcerative colitis (UC) showed that there was an 11% decrease in the density of P2X7R immunoreactive neuronal cell bodies (da Silva et al., 2015). Much of the recent research that has explored the P2X7R in IBD has also studied the role of connexins and/or pannexins in ATP release. This will be discussed later in the present review.

### Other purinergic receptors in inflammatory bowel disease

Although the P2X7R appears to play a key role in IBD, other purinergic receptor subtypes may also contribute to or be affected by IBD pathophysiology. Guzman et al. observed an upregulation of mRNA for P2X1R, P2X4R, P2X7R, P2Y2R, and P2Y6 receptors in a rat model of TNBS-induced colitis (Guzman et al., 2006). A later bioinformatics study found that genetic dysregulation in IBD was observed in 59% of purine genes such as P2Y5R, P2Y6R, P2Y13R, P2Y14R, and P2X5R (Rybaczyk et al., 2009). The P2X3R was observed to have changes in expression of mRNA in both patients with CD or with UC, where immunoreactivity on the enteric ganglia of IBD was increased (Yiangou et al., 2001). A more recent study observed a downregulation in immunoreactivity of P2X3R in enterochromaffin cells of specimens from UC patients which may influence the autocrine regulatory role of ATP that is involved in the release of 5-HT from these cells (Linan-Rico et al., 2013; Patel, 2014). In another preclinical study using TNBS-induced colitis in mice, protein expression of P2X1R was upregulated in the submucosa of colitis tissue and degradation of ATP by nucleotidases was increased during colitis (Lomax et al., 2007). The role of nucleotidases will be discussed further below.

### Role of ectonucleotidases in inflammatory bowel disease

Ectonucleotidases are responsible for the degradation of ATP once released and are found to be significantly altered in IBD (Antonioli et al., 2013). In a DSS mouse model of colitis, CD39 deletion was shown to exacerbate colitis (Friedman et al., 2009). In the same study, it was shown in humans that single nucleotide polymorphisms with low CD39 expression were associated with CD (Friedman et al., 2009). CD patients have also been observed to have lower CD39 expression in Th17 cells (Longhi et al., 2014). In addition, UC and CD patients undergoing remission after treatment from anti-tumor necrosis factor therapy were found to have elevated CD39 expression on T-regulatory cells (Gibson et al., 2015). CD73 has been studied in the context of a CD73^−∕−^ mouse DSS colitis model, where exacerbated inflammation of DSS colitis was observed in the knockouts (Bynoe et al., 2012). The changes observed in these ectonucleotidases may influence purinergic receptor activation; for instance, it has been demonstrated that a knockout of CD39 reduced P2X7R responses in mast cells (Kuhny et al., 2014). Increases in ATP levels associated with a downregulation of ectonucleotidases may exacerbate P2XR activation and inflammation in other cells.

## Connexins and their roles in ATP release and inflammation

Connexin gap junctions and hemichannels are expressed throughout different types of mammalian tissues. Within the intestine, the range of connexins includes the following: Cx26, Cx32, Cx36, Cx37, Cx43, and Cx45 (Wang and Daniel, 2001; Kanczuga-Koda et al., 2004; Morita et al., 2004; Clair et al., 2008; Ezumi et al., 2008; Frinchi et al., 2013).

### Cx43 hemichannels

The most studied subtype of connexins in the context of intestinal inflammation and motility is Cx43. The Cx43 hemichannel has been shown to open to allow for the release of ATP, which has been demonstrated in many cell types such as Cx43 transfected C6 cells and polymorphonuclear leukocytes (Eltzschig et al., 2006; Kang et al., 2008). Cx43 is proposed to be involved in microglial survival where ATP release is modulated by Cx43 hemichannel opening (Ma et al., 2014). In macrophages, it has been suggested that Cx43 is colocalized with P2X7R and that together they mediate intercellular communication via gap junction formation that is influenced by extracellular ATP (Fortes et al., 2004). In one study which further emphasizes its role in inflammation, Cx43 was suggested to be the conduit for extracellular release of prostaglandin (PG) E_2_ (Cherian et al., 2005). Interestingly, in another study PGE-ethanolamides and PGF-ethanolamides were shown to be protective against tissue damages in a human explant colitis model induced by TNFα and IL-1β (Nicotra et al., 2013). Cx43 has been linked to inflammation of the intestine and diarrhea due to bacterial infection, where an increase of Cx43 was linked to increased occurrence of bacterial infection in colonocytes (Guttman et al., 2010; Vinken et al., 2010). Cx43 has been implicated in facilitating internalization of bacteria and overall mucosal integrity (Velasquez Almonacid et al., 2009; Bou Saab et al., 2014). Studies that have looked at Cx43 in the context of gut motility have focused on Cx43 expression on smooth muscle and enteric ganglia and its role in calcium wave propagation. In one study using transgenic mice with conditional deletion of Cx43 in intestinal smooth muscle, there was a 29% decrease in GI transit time (Doring et al., 2007). Intracellular calcium has been suggested to regulate Cx43, where a lack of Cx43 delayed overall colonic transit time (Lurtz and Louis, 2007; McClain et al., 2014). Thus, the abnormality of connexins, particularly Cx43, may be involved in both intestinal dysmotility and inflammation that is associated with IBD and other intestinal conditions.

### Cx32, Cx36, Cx37, and Cx45 hemichannels

Cx32 hemichannels have also been suggested to release ATP in response to increases in intracellular calcium ions in transfected C6 glioma cells (De Vuyst et al., 2006). Cx36 is expressed in mouse myenteric ganglia, and Cx36 in rat neuron cultures was suggested to release ATP during depolarization of neurons (Schock et al., 2008; Frinchi et al., 2013). In a study by Koutsoumpas et al. the presence of autoantibodies for Cx37 was determined in a cohort of CD patients, suggesting CD patients developed an autoimmunity to resident Cx37 and thus may be involved in IBD pathogenesis (Koutsoumpas et al., 2011). In mongrel dogs, varying levels of immunoreactivity for Cx45 were observed along the GI tract, with some Cx45 protein present on myenteric and submucosal gangliaand sparse immunoreactivity on the circular muscle of the esophageal sphincter and ileum (Wang and Daniel, 2001).

## Pannexin channels and their involvement in inflammation

Pannexin channels are relatively newly identified ATP release channels. They were discovered based on their sequence similarity to innexins, the gap junction proteins of invertebrates (Baranova et al., 2004). Among the three types of pannexin in mammals, Panx1 channels are ubiquitous in mammalian tissues and have an extensive list of functions that have been ascribed to them. Panx2 proteins are localized to the central nervous system and more recently have been found in enteric neurons (Baranova et al., 2004; Lai et al., 2009; Swayne et al., 2010; Li et al., 2011; Diezmos et al., 2015). Panx3 is expressed in skin and cartilage, and has been suggested to act as an ATP release channels in these tissues (Bruzzone et al., 2003; Ishikawa et al., 2011). Pannexins do not form gap junctions and exist solely as cell membrane channels (D'Hondt et al., 2009). Their role as ATP release channels was reported soon after their discovery. Under stimuli such as mechanical stress, the channel can open to release intracellular ATP into the extracellular space which in turn can act on P2XR or P2YR in the vicinity (Locovei et al., 2006b; Xia et al., 2012; Beckel et al., 2014).

Panx1 has been shown to be involved in ATP release in different models of inflammation. Pharmacological blockade of Panx1 was shown to decrease IL-1β release and caspase-1 production in J774 macrophages (Pelegrin and Surprenant, 2006). Other studies have investigated T-cells in relation to antigen presentation, where Panx1, P2X1R, and P2X4R were found to mediate calcium entry into the T-cells and IL-2 production (Woehrle et al., 2010a). These events occur as a result of the translocation of Panx1, P2X1R, and P2X4R toward the “immune synapse,” a complex formed during T cell activation (Woehrle et al., 2010a). Another study found that T-cell receptor activation leads to calcium ion influx, which causes the extracellular release of ATP via Panx1 channels (Schenk et al., 2008). T-cells were also suggested to participate in cell death that involves Panx1 channels acting downstream of activated P2X7R (Shoji et al., 2014). In the Jurkat T-cell line, Panx1 was found to be crucial in regulating fragmentation of apoptotic cells (Poon et al., 2014). In addition, Panx1 was shown to mediate release of nucleotides from apoptotic cells which signals for the recruitment of phagocytes (Chekeni et al., 2010). Panx1 expressed in neutrophils is suggested to function in conjunction with P2Y2R in an autocrine fashion to mediate chemotaxis (Bao et al., 2013).

Expression of Panx1 and purinergic receptors in a range of immune cells has thus led to research into their roles in various inflammatory diseases. P2X7R and Panx1 are shown to participate in neutrophil activity and recruitment, as well as IL-1β release in a study focusing on chronic obstructive pulmonary disease (COPD) (Riteau et al., 2010). Blockade of Panx1 function attenuated the elevation of ATP levels normally observed in COPD (Baxter et al., 2014). A recent study in mice has suggested that P2X7R is involved in brain ischemia alongside Panx1 and together are thought to be involved in a neuroprotective mechanism of ischemia pre- and post-conditioning, and in improved memory and motor functions in mice with ischemia-reperfusion-induced cerebral injury (Mahi et al., 2015). In an autoimmune encephalomyelitis mouse model for multiple sclerosis, P2X7R is upregulated during the chronic phase of the disease (Lutz et al., 2013). Blockade of functioning Panx1 channels reduced clinical signs of encephalomyelitis (Lutz et al., 2013). Panx1^−∕−^ and Panx2^−∕−^ double knockout mice were more resistant to ischemic stroke, which was not observed in single Panx1^−∕−^ or Panx2^−∕−^ knockouts (Bargiotas et al., 2011). A further study demonstrated that exploration, sensorimotor functions, anxiety, and behavioral symmetry in the double knockout mice were improved (Bargiotas et al., 2012).

The aforementioned disease models thus have the following in common with IBD: infiltration of neutrophils and other immune cells, changes in cytokine release, and alterations in either pannexin and/or purinergic receptors. Similar to connexins, pannexins are expressed in the ENS within enteric ganglia and other cells involved in the immune response, making pannexins potential players alongside connexins in ATP associated dysmotility and inflammation (Gulbransen et al., 2012; Diezmos et al., 2013, 2015). Panx1 expression was found to be reduced in the ENS of CD patients but not in UC patients (Gulbransen et al., 2012). In contrast, immunohistochemistry results have shown that Panx1 immunoreactivity on human colonic tissues was almost abolished in myenteric ganglia of specimens from UC patients but not in those from CD patients (Diezmos et al., 2013). Interestingly, the decrease in Panx1 protein expression in CD as observed by Gulbransen and colleagues was also demonstrated by Diezmos et al. using Western blot analysis (Gulbransen et al., 2012; Diezmos et al., 2013). Panx2 has also been examined in human colonic tissues where up-regulation of Panx2 mRNA was found in the muscularis layer of UC specimens, though this was not reflected at the protein level or immunoreactivity (Diezmos et al., 2015). The limited and conflicting results presented here warrant further studies in this area; specifically to confirm the true expression profile of Panx1 in normal intestine and IBD tissues, to determine the extent in which pannexins and connexins contribute to extracellular ATP signaling in IBD pathophysiology, and to map the process of events leading to inflammation.

## Interactions between connexins, pannexins, and purinergic receptors in inflammatory bowel disease

Currently, there are a handful of studies that have examined the interactions between connexins, pannexin, and purinergic receptors. Of these studies, only a few have looked at these proteins in the context of IBD. The study conducted by Gulbransen et al. was the first to look at Panx1 and P2X7R in IBD, examining expression and function in a mouse model of colitis and in human samples from UC and CD patients (Gulbransen et al., 2012). In a mouse model of colitis it was shown that inhibition of P2X7R, Panx1, the adaptor protein apoptosis-associated speck-like protein containing CARD (ASC) or caspase activity attenuated the inflammation process that caused enteric cell death (Gulbransen et al., 2012).

The localization of connexins, pannexins, and purinergic receptors within the gut regions and tissues may provide insight into their specific roles in inflammation. Panx1 expression is ubiquitous and is found on glandular epithelium, non-lymphoid leukocytes, blood vessel endothelium, erythrocytes, varicosities within ganglia, and muscle tissue (Diezmos et al., 2013). Panx2 is localized to mast cells, mucosal epithelial cells, non-lymphoid leukocytes, smooth muscle tissue, as well as myenteric and submucosal ganglia where Panx2 is co-localized with β-tubulin, substance P, neuronal nitric oxide synthase, calcitonin gene-related peptide, and vesicular acetylcholine transporter (Diezmos et al., 2015). Cx43 is found throughout the smooth muscle layer, on interstitial cells of Cajal, and on the cell bodies and processes of enteric ganglia (Nemeth et al., 2000; McClain et al., 2014). As mentioned previously, P2X7R is expressed on submucosal and myenteric ganglia (Gulbransen et al., 2012; Kurashima et al., 2012).

**Figure 1:**
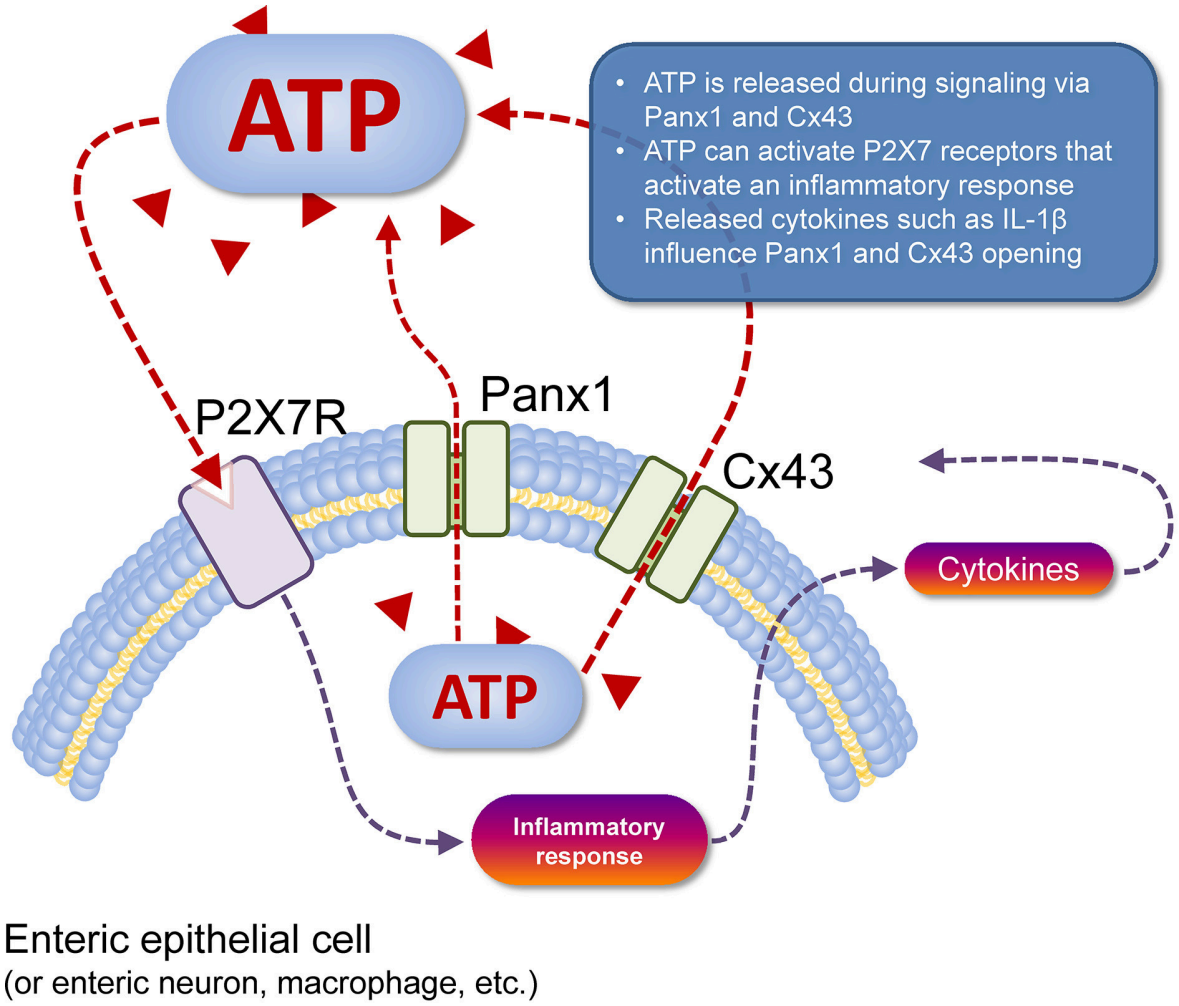
**Cell type-specific schema of ATP release and action**. ATP (red triangles) can be released from the cell cytosol to the extracellular space (dashed red line) via Panx1 channels or Cx43 hemichannels (pictured). Once in the extracellular space, this ATP acts as a paracrine transmitter, as can ATP released from nearby cells that are dead or dying (not shown). Extracellular ATP can activate P2 receptors, such as P2X7R (pictured) that depolarises the target cell, but also activates an inflammatory response in immune cells (dashed gray line) with subsequent release of cytokines such as IL-1β that can act back at Panx1 and Cx43 to modulate their function (dashed gray lines). Activation of P2X7R also mediates the T-cell response (e.g., Ca^2+^ entry, IL-2 synthesis) and macrophage migration (not shown).

The current consensus is that ATP is released from Panx1 channels and possibly Cx43 hemichannels. This ATP functions in an autocrine and/or paracrine manner in conjunction with P2X7R (Figure 1). In the context of inflammation, activation of the Panx1/P2X7R complex can lead to the formation of the NACHT, LRR, and PYD domains-containing protein 3 inflammasome involving caspase-1 which in turn leads to release of mature IL-1β (Surprenant and North, 2009). The mechanism by which this occurs however is unknown (Surprenant and North, 2009). Panx1 was suggested to regulate ATP release which in conjunction with P2X1, P2X4, and P2X7 mediates T-cell responses such as Ca^2+^ entry and IL-2 synthesis (Woehrle et al., 2010a,b). In astrocytes, Panx1 expression is required for the release of IL-6, IL-8, and glutamate (Wei et al., 2016). Interestingly, Cx43 in astrocytes were shown to open in response to IL-1β and TNF-α release from microglia (Retamal et al., 2007). Panx1 may also have an important role in cell migration. Panx1 and P2X7R via different mechanisms are required for GM-CSF promoted macrophage fusion (Lemaire et al., 2011). A more recent study has shown that activation of vascular endothelial cells by TNF-α causes Panx1-mediated ATP release, leading to leukocyte migration during inflammation (Lohman et al., 2015).

UC and CD can be differentiated by the cytokine profile exhibited during disease (Khor et al., 2011). UC is thought to be Th2 cell mediated and is associated with elevated IL-5 levels and a lack of IL-2 or IFN-γ increase (Fuss et al., 1996; Sanchez-Munoz et al., 2008). CD on the other hand is suggested to be Th1 and Th17 cell mediated with elevated IFN-γ and IL-22 (Fuss et al., 1996; Monteleone et al., 1997; Plevy et al., 1997; Sanchez-Munoz et al., 2008; Strober and Fuss, 2011). Common to both UC and CD inflammation are elevated levels of TNFα, IL-1β, IL-6, IL-8, IL-12, and IL-17 (Fiocchi, 1998; Papadakis and Targan, 2000; Sanchez-Munoz et al., 2008; Strober and Fuss, 2011). Thus, the altered expression of cytokines observed in IBD may be linked to the differential expression of Panx1 and Cx43 that is seen between certain cell types.

Most studies have concluded that Panx1 is the more important channel in mediating ATP release and in conjunction with P2X7R is important in cytokine release such as IL-1β (Pelegrin et al., 2008; Iglesias et al., 2009; Lemaire et al., 2011; Thi et al., 2012; Orellana et al., 2013; Baxter et al., 2014; Kanjanamekanant et al., 2014; Ohbuchi et al., 2014; Poon et al., 2014; Shoji et al., 2014). Nonetheless, at least three studies have concluded that Cx43 as the more important channel in ATP release and inflammation particularly in enteric glia (Decrock et al., 2009; Csoka et al., 2015; Brown et al., 2016). Finally, some studies have concluded that neither Cx43 nor Panx1 have a significant influence on ATP release (Kurashima et al., 2012; Avendaño et al., 2015). In some cases, P2X7R activation is independent of their expression (Alberto et al., 2013; Hansen et al., 2014). Thus, it is still difficult to determine whether Panx1 and/or Cx43 will play a more influential role in the pathophysiology of IBD if one does indeed exist. Table 2 summarizes a range of articles that have determined whether Panx1 or Cx43 is more important for ATP release.

**Table 2:** **Literature focusing on cell type-specific function of Cx43, Panx1, and P2X7R**.

**Study**	**Channel/receptor focused on in study**			**Context**
	**Connexin-43**	**Pannexin-1**	**P2X7 receptor**	
Alberto et al., 2013	• Studied • Not important for ATP release	• Studied • Not important for ATP release	• Studied • Dye uptake independent of Cx43 and Panx1	Murine macrophages
Avendaño et al., 2015	• Studied • Increased channel opening	• Studied • Increased channel opening	• Studied	Prenatal exposure to inflammation
Baxter et al., 2014	• Studied	• Studied^*^ • ATP release attenuated with channel blocker	• Studied	Airway epithelia
Brown et al., 2016	• Studied^*^ • Responsible for majority of ATP release	• Studied	• Studied	Enteric glia
Csoka et al., 2015	• Studied^*^ • Determined to be more important for ATP release	• Studied	• Studied	Murine macrophages in sepsis
Decrock et al., 2009	• Studied^*^ • Contributes to apoptosis	• Studied Not detected	• Studied	Rat C6 glioma cells
Gulbransen et al., 2012	• Not studied	• Studied • Important for mediating cell death	• Studied	Enteric neurons
Hansen et al., 2014	• Studied • Membrane conductance not observed	• Studied • Dye uptake independent of P2X7R	• Studied • Dye uptake independent of Panx1	Xenopus laevis expression system
Iglesias et al., 2009	• Studied • Does not form cell membrane channel	• Studied^*^	• Studied	Astrocytes
Kanjanamekanant et al., 2014	• Studied • Not deemed important	• Studied^*^ • Increase in release of IL-1β and ATP	• Studied	Human periodontal ligament cells
Kurashima et al., 2012	• Studied • Not important for ATP release	• Studied • Not important for ATP release	• Studied	Mast cells
Lemaire et al., 2011	• Studied • Gap27 blocker had no effect	• Studied^*^ • Concluded to be an important channel	• Studied	Rodent multinuclear macrophages
Ohbuchi et al., 2014	• Studied	• Studied^*^ • Blockage of channel inhibited ATP release	• Studied	Human airway epithelia
Orellana et al., 2013	• Studied • No effect on ATP release when inhibited	• Studied^*^	• Studied	Astrocytes
Pelegrin et al., 2008	• Not Studied	• Studied^*^ • Important for IL-1 release	• Studied	Peritoneal mouse macrophages
Poon et al., 2014	• Studied	• Studied^*^ • Important for cellular disassembly	• Not studied	Jurkat cells and Thymocytes from mice
Schenk et al., 2008	• Studied	• Studied^*^ • ATP release reduced when channel was blocked	• Studied	Activated T cells
Shoji et al., 2014	• Studied	• Studied^*^	• Studied • Overexpressed in T cells of KO mice	Mouse T cells
Thi et al., 2012	• Studied	• Studied^*^ • Concluded to play a bigger role	• Studied	Osteoblasts

* *Specifies the more important or significant channel in the study*.

## Pharmacological tools and knockout mice

The pharmacological tools available in the current field of pannexin and connexin research have gradually improved with the availability of more selective channel blockers. However, there is still a lack of high affinity inhibitors for most pannexins and connexins. For connexins, both gap junction and hemichannel activity needs to be considered (Verselis and Srinivas, 2013). Carbenoxolone has been used as a connexin channel blocker but has since been shown to block Panx1 channels and alter network activity of cultured neurons (Rouach et al., 2003). The Cx43 mimetic peptide 5 and peptide Gap19 selectively block Cx43 hemichannels but not gap junctions or other connexin hemichannels (O'Carroll et al., 2008; Wang et al., 2013b). For pannexin channels, only selective Panx1 channels blockers exist such as mefloquine, probenecid and ^10^Panx1 (Good et al., 2015). However, these tools are not selective enough to determine underlying Panx1 channel mechanisms due to additional effects on proteins such as connexins (Good et al., 2015). In contrast, the pharmacological tools for P2 receptors include selective antagonists for many receptors. For example, the specific receptor antagonists A-438079 and A-740003 have been developed for P2X7R (Donnelly-Roberts and Jarvis, 2007).

A selection of knockout mice for connexins, pannexins and P2X7R has been generated in previous studies. Pannexins and connexins are generally well-conserved between humans and mice. Pannexins show 93-94% conservation at the protein level (Penuela et al., 2009). Likewise, Cx43 and Cx45 show 98% conservation at the genetic level, making mice a translatable animal model (Sohl and Willecke, 2004). Panx1 knockouts have been previously explored in another review article, showing that there were no major phenotypic abnormalities in the knockout mice despite wide-spread expression of Panx1 (Penuela et al., 2013). Panx2^−∕−^ knockout mice have been generated and did not show major differences in phenotype compared to wild type mice (Bargiotas et al., 2011). However as mentioned above, Panx1^−∕−^ and Panx2^−∕−^ double knockout mice were shown to have resistance to ischemic stroke (Bargiotas et al., 2011). The development of Panx3 knockout mice has recently been published and mice lacking the Panx3 gene were shown to be less susceptible to osteoarthritis development (Moon et al., 2015). Connexin knockouts have previously been reviewed in the context of cardiac function, showing that a large number of connexin knockouts are lethal (Lo, 2000). The Cx43^+∕−^ heterozygous knockout mice are able to survive adulthood but show a ventricular conduction phenotype (Guerrero et al., 1997). As mentioned previously, mice with a conditional knockout of Cx43 in GI smooth muscle showed decreased motility (Doring et al., 2007). In P2X7R knockout mice macrophages were found to lack the ability to release IL-1 (Solle et al., 2001).

Using combinations of the available pharmacological tools and knockout mice hold promise in the progression of research in this field. The hope is to confirm the specific roles of pannexins and connexins in ATP release and in disease pathophysiology. This task is made difficult by the still weak selection of pharmacological tools available and the potential problems of compensation by other subtypes in gene silencing and knockout models. For instance, Panx3 is not normally expressed on blood vessels but is significantly upregulated in the arterial wall of Panx1 knockout mice (Lohman and Isakson, 2014).

## Conclusions and future directions

The role of pannexin and connexin channels in gut inflammation is an emerging and exciting topic of research. Such research will be useful in determining the etiology of IBD, which is yet to be fully understood. Future studies that examine the extent of pannexin and connexin channel activation would provide valuable knowledge in determining the involvement of P2X7R and other purinergic receptors in the GI system, in both health and disease states, and in defining the potential interactions of Panx1, Cx43 and P2X7R in IBD.

## Author contributions

ED wrote the manuscript and conducted the research, PB conducted the research and assisted with the writing and editing of the manuscript, LL conducted the research and the editing of the manuscript.

### Conflict of interest statement

The authors declare that the research was conducted in the absence of any commercial or financial relationships that could be construed as a potential conflict of interest.

## Abbreviations

CD: Crohn's disease
COPD: chronic obstructive pulmonary disease
Cx: connexin
DSS: dextran sulfate sodium
ENS: enteric nervous system
GI: gastrointestinal
IBD: inflammatory bowel disease
Panx1: pannexin-1
Panx2: pannexin-2
Panx3: pannexin-3
P2XR: P2X receptors
P2YR: P2Y receptors
P2X7R: P2X7 receptors
TNBS: trinitrobenzene sulfonic acid
UC: ulcerative colitis.
